# An IoMT-Based Melanoma Lesion Segmentation Using Conditional Generative Adversarial Networks

**DOI:** 10.3390/s23073548

**Published:** 2023-03-28

**Authors:** Zeeshan Ali, Sheneela Naz, Hira Zaffar, Jaeun Choi, Yongsung Kim

**Affiliations:** 1 R & D Setups, National University of Computer and Emerging Sciences, Islamabad 44000, Pakistan; 2 Department of Computer Science, COMSATS University Islamabad, Islamabad 45550, Pakistan; 3 Department of Computer Science, Air University, Aerospace and Aviation Kamra Campus, Islamabad 44000, Pakistan; 4 College of Business, Kwangwoon University, Seoul 01897, Republic of Korea; 5 Department of Technology Education, Chungnam National University, Daejeon 34134, Republic of Korea

**Keywords:** computer-aided design, medical assistance, skin cancer, melanoma lesion, generative adversarial networks, survival rate, Internet of Medical Things (IoMT)

## Abstract

Currently, Internet of medical things-based technologies provide a foundation for remote data collection and medical assistance for various diseases. Along with developments in computer vision, the application of Artificial Intelligence and Deep Learning in IOMT devices aids in the design of effective CAD systems for various diseases such as melanoma cancer even in the absence of experts. However, accurate segmentation of melanoma skin lesions from images by CAD systems is necessary to carry out an effective diagnosis. Nevertheless, the visual similarity between normal and melanoma lesions is very high, which leads to less accuracy of various traditional, parametric, and deep learning-based methods. Hence, as a solution to the challenge of accurate segmentation, we propose an advanced generative deep learning model called the Conditional Generative Adversarial Network (cGAN) for lesion segmentation. In the suggested technique, the generation of segmented images is conditional on dermoscopic images of skin lesions to generate accurate segmentation. We assessed the proposed model using three distinct datasets including DermQuest, DermIS, and ISCI2016, and attained optimal segmentation results of 99%, 97%, and 95% performance accuracy, respectively.

## 1. Introduction

The Internet of Things (IoT) has expanded into many research domains such as smart cities, vehicular communication, cloud computing, smart agriculture, and healthcare systems [1,2,3]. However, the research interest has mainly been focused on the healthcare systems of IoMT-based medical systems. IoMT-based medical systems are based on sensors or smart medical devices for data collection, and they also perform cloud-based data processing [4]. These systems are used for virtual patient care or long-term illness monitoring as well as patient medication tracking. By linking patients to respective physicians as well as allowing healthcare data to be exchanged over a secure site that could be anywhere, the Internet of Things substantially lowered the number of needless hospitalizations [5]. Using IoMT technology, patients and diagnostic laboratories may access data online from anywhere and at any time [6]. IoMT-assisted techniques bring significant advances to a range of medical fields that need rigorous study and supervision, including early diagnoses, such as diabetes [5], heart disease [7], infectious diseases [8], as well cancer diseases [9]. These diseases are detected early on and tracked utilizing IoT-based medical technologies. Due to these advantages, IoMT-based technologies play a remarkable role in the medical sector by designing effective Computer-aided diagnostic (CAD) systems. Similarly, in dermatology, it is critical to detect skin lesions in the very beginning phases to prevent subsequent consequences including melanoma. Annually, about nine thousand people die due to skin cancer. Of all the kinds of skin cancer, about 2% are melanoma, which causes 75% of deaths from skin cancer [10]. To diagnose the medicinal conditions of melanoma lesions, dermatologists visually analyze the symmetry, irregularities in edges, color differences, and diameter of more than 6 mm. All of these are indications of skin cancer, commonly called the ABCDE rule, proposed by the American Society for cancer [11]. On the other hand, the performance accuracy of the manual diagnosis of this rule is around 59~80% [12]. However, performance accuracy only depends on the test called ‘biopsy’ [13]. The diagnosis of melanoma is looked for at its initial phases because it can be cured at this early phase through excision of the affected lesion. Manual diagnosis, on the other hand, first requires an expert dermatologist, and then, in the second step, the decision is sent to for a subjective variation analysis. All of these manual diagnoses take time and may be risky since patients’ lives are in jeopardy [14].

Therefore, to facilitate the patients as well as dermatologists, automatic diagnosis is significant. It is a reliable procedure that also saves time by reducing the series of biopsies tests. The segmentation of skin lesions is crucial in the performance of diagnostics in these automated CAD systems of skin disease diagnostics. Segmentation is an important task to analyze medical images by dividing the pixels of images into appealing regions based on various characteristics including texture, grey level, color, as well as intensities of pixels. Generally, in the CAD system, the underlying modalities, such as a dermoscopic image, are employed to identify skin cancer. In this scenario, accurate segmentation is critical because segmentation localizes the lesion regions, and by using these localized areas, the detector model will result in a more ideal classification of skin cancer, such as melanoma or benign. Poorly located regions or faulty segmentation will result in incorrect disease diagnosis and analysis. Hence, in existing studies, the dermoscopy images approach is utilized to deeply examine the lesion in terms of segmentation and to improve its diagnosis [15]. These images visually enhance the clarity to a certain level, but an accurate diagnosis of melanoma continues to be challenging due to the size of the lesion and arbitrary shape. In addition, variations in hue, edges, form, and noise such as hair and air bubbles also cause difficulty to segment out the lesion. Furthermore, the contrast between the lesion and the surrounding area is low, which also hinders precise segmentation. Numerous researchers have proposed some traditional approaches such as Otsu [16] and Stochastic [17] to segment out melanoma, but all of these thresholding algorithms are not automated and suffer from the problem of under or over-segmentation due to artifacts.

Recent studies have used deep learning-based frameworks in medical applications for melanoma segmentation and detection. However, most research has already taken significant advances toward automating analysis by employing CNN in a variety of image recognition studies [18]. In the case of CNN, it performs automatic feature learning after receiving image data by utilizing various loss functions and parameters based on the task. For instance, we take the naive technique for minimizing the Euclidean distance between predicted and ground truth by initializing CNN. At first, CNN will start producing blur images [19,20] because Euclidean distance can only be minimized by taking the average of all possible outputs. In terms of the loss function, forcing CNN to do what we want is an open problem and requires domain experts. In all of these existing methods, the main problem in melanoma lesion segmentation is the challenge of visual similarities between melanoma lesions and normal skin. Despite dermoscopic images’ increased sharpness, accurate melanoma localization from dermoscopic images remains difficult due to broad variance in color, texture, structure, and the presence of artifacts such as hair, gel bubbles, and clinical rule markings.

Recently, generative deep learning models, notably Generative Adversarial Networks (GANs), have been applied in a number of applications for a variety of tasks. A GAN trains a generative model to minimize the loss after learning the loss associated with classification attempts [16,21]. In the literature context of melanoma lesion segmentation, several methods are proposed, which include traditional methods such as Premaladha [22], thresholding-based [23], etc.; however, these methods fail when some diverse changes are observed in melanoma lesions images since these are parametric methods that are set manually. Recently, deep learning-based methods are widely used for classification and segmentation. The segmentation models include U-Net, FCN, and many more. However, the evolution of generative models opens up new avenues for solving diverse problems. As a result, one key research issue arises: what if these generative models are employed to perform segmentation? Second, the segmented images must be generated from specific dermoscopy images in which the lesion is emphasized, which is why conditional GANs are one of the best variants [21]. In CGANs, the images are generated depending on a Condition. This characteristic makes the cGAN pix-to-pix translation task where we can apply conditions on an input image and can generate its corresponding output images. There are some GAN-based approaches for skin lesion segmentation in existing studies, and the types of GANs used in these studies vary. Some have utilized style-based GANs [24], some have utilized them for augmentation [25], some have utilized traditional deep Generative adversarial networks (DGANs) [26], etc. Here, one major research question arises, what if skin lesions are segmented by structuring the problem in such a manner that generative models generate the segmented images based on the conditions? To answer this question, we have suggested conditional GANs for the task of skin lesion segmentation. Moreover, the major reason behind using cGANs is to assess the performance of segmentation tasks by using more recent and advanced deep learning models in comparison with existing traditional methods especially the melanoma lesions with cGANs. In addition, we also performed the preprocessing on dermoscopy images to enhance them to be more effectively utilized by cGANs. Thirdly, we performed the experimentation on three different datasets having a diverse range of skin lesion images to properly analyze the performance of CGANs for skin cancer lesion segmentation tasks. Our contribution is summarized in the following points:

- Conditional Generative Adversarial Networks (CGANs) are suggested to carry out the task of melanoma lesion segmentation and multiple types of cancer from a single image.
- Skin refinement as a preprocessing step is employed to automatically remove the artifact from images.
- The proposed segmentation technique accurately segments the affected lesion by overcoming the challenges presented in the ISIC2016, DermIS, and DermQuest datasets.

The rest of the paper is organized in the following way: Section 2 thoroughly explains the current approaches. Section 3 explains our proposed methodology in detail. Section 4 explains the experimental detail and results. Lastly, Section 5 provides the conclusion of the paper.

## 2. Related Work

Melanomic segmentation is a critical method for improving accuracy when developing automated skin cancer detection algorithms. Approaches to automatic segmentation are further classified as classical methods and deep learning methods. The traditional methods mainly include iterative selection [27,28], adaptive threshold [23], iteration merging of regions [17], and the Otsu threshold [29]. However, the performance of threshold-based segmentation approaches is affected due to the presence of artifacts in images [27,29]. In [29], the performance of the study is noticeable, but segmented image boundaries are irregular and reduce image resolution. In [30], the researchers overcame the limitations that are exhibited in [29]. Another group of researchers [17,31] applied a region-merging approach for segmentation. This approach groups the intensely similar regions of the image. To reduce the effects of hues, contrast, and illustration, the region-merging approach is effective. In [17], these similar attributes were utilized for the segmentation of lesions. In [32], for image segmentation, an active contour model governed by an adaptive local pre-fitting energy function relying on Jeffreys divergence is presented. When contrasted with local region-based algorithms as well as previously developed algorithms, their suggested method not only reduces computing costs significantly but also enhances segmentation results. Such active contour models are also utilized in existing studies as a post-processing stage after the segmentation results of the melanoma lesions [33]. These active contour models in conjunction with morphological operations are also utilized as a post-processing step [34]. Moreover, Fuzzy-C means-based clustering is also integrated with active contour models to further enhance skin lesion segmentations [35]. These traditional methods perform well; however, these are parametric methods that require multiple parameters to be manually specified to achieve excellent segmentation, such as thresholding-based methods.

Deep learning-based methods have recently been widely adopted in practically all application domains [36,37,38,39]. They are also very much applicable in medical imaging domains [40,41,42]. For instance, in [43], a deep learning method based on transfer learning is proposed for the classification of skin lesions from images. In this study, automated features are retrieved from images utilizing several pre-trained models such as VGG19, Inception V3, ResNet50, and SqueezeNet, and the best results are obtained. Rather than focusing just on improving feature extraction, feature selection is also considered to increase performance. For instance, in [44], several feature selection methods are proposed such as gradient boosting, statistical methods, and optimization algorithms such as PSO. Likewise, in [45], the deep learning method by using U-Net along with stochastic weighted averaging for the segmentation task of melanoma is suggested. When compared to existing approaches, their suggested technique achieves both high precision and real-time efficiency. Subsequently, in some studies, more challenging skin cancer lesion images are utilized to evaluate the performance of the algorithms [46].

In addition to the aforementioned literature, several advanced deep learning methods, such as Generative adversarial networks (GAN), are now being used in the majority of medical imaging applications. For instance, in [47], adversarial training and deep transfer learning-based methodology are proposed to automate the detection of melanoma detection. Their trained model minimizes the focal loss function, which aids the model in optimal learning from difficult samples while underweighting the simple ones. Similarly, in [26], the skin lesion classification method based on GAN with an improved set of hyperparameters is proposed to overcome the problem of network instability. In addition, in [26], the GAN-based method is utilized for the task of augmentation. They are also utilized in an unsupervised manner for different tasks related to skin cancer lesions such as removing noise from the images as well as other artifacts [48]. Current studies have utilized the cGAN on some discrete labels [16,49,50] text [51], and images as well. Image-conditional frameworks undergo the prediction of images from future maps [52], normal maps [53], generation of images from sparse annotation [54,55], and generation of the product photo [56]. By forcing the output to be conditional on the corresponding input, some studies have utilized GAN and attained high results in style transfer [57], impainting [19], super-resolution [57], and manipulation of images [58].

To diagnose the affected region in dermoscopic images, some approaches [59,60] have applied object-detection algorithms. The hyper-graph technique was utilized in [59] to map saliency by utilizing super-pixel information. Other researchers [14,15,61,62] utilized deep learning-based approaches for segmentation and attained significant results as compared to traditional approaches. In [14], an FCRN, i.e., fully-convo-residual network, was proposed to overcome the overfitting issue in segmenting melanoma. In [15], region-based CNN was utilized to localize the lesion and then utilize the machine learning fuzzy clustering approach.

Moreover, in [61], a 19-layer CNN is designed to enhance the performance accuracy by segmentation of melanoma. In their research, they used Jaccard distance as a loss function. This loss function overcomes the overfitting issue between melanoma and normal skin images, consequently improving performance accuracy. In [62], FRCN, i.e., full-resolution CNN, was proposed to segment melanoma. This CNN learned segmentation of the model through a full image without applying any preprocessing to the dermoscopy images. In [63], to segment melanoma and reduce the effect of artifacts, a hybrid approach was introduced with the combination of Convolutional and Recurrent networks. However, two-phased object detection frameworks such as RCNN generate approximately two thousand regions for each image to estimate melanoma lesions.

As compared with the other approaches, the proposed approach of cGAN is different. We have used U-Net-centered architecture [64] for our generator and utilized a convolutional Patch GAN classifier for the discriminator. For purpose of local statistical analysis, a very similar Patch GAN architecture was utilized in [57].

## 3. Materials and Methods

**Figure 1:**
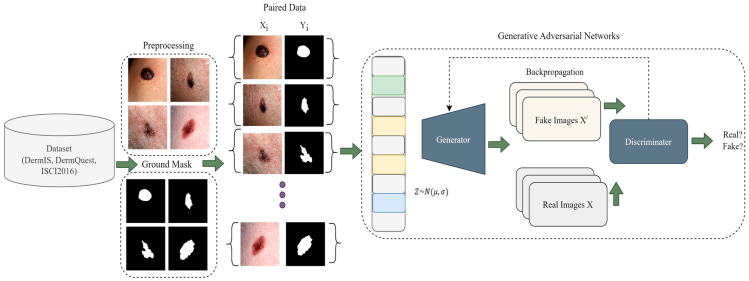
A Pictorial Overview of Proposed Methodology.

In this section, we go over the proposed work step by step. To summarize, the steps of the proposed framework begin with data preprocessing, followed by data augmentation, and finally, using the processed images to train the CGANs model. More precisely, we begin by showing how the melanoma lesion segmentation task can be handled using CGANs, and then in the next part, we outline the problem in more thorough steps, before explaining the internal architectural details and training of the CGANs. In the last, the hyperparameters of the model are also discussed. The main overview of the proposed methodology is depicted in Figure 1.

GAN is a generative model and it learns how to map using a random noise vector z to output images y, G:z→y [21]. On the other hand, conditional GAN is a model that learns mapping by observed images x and random noise-vector z, to y, $G:\;\left\{x,z\right\}\to y$. The G, i.e., generator, is trained to produce output that cannot be distinguished from an original image by D, i.e., an adversarially trained discriminator, which is trained to classify real and fake images.

### 3.1. Preprocessing

**Figure 2:**
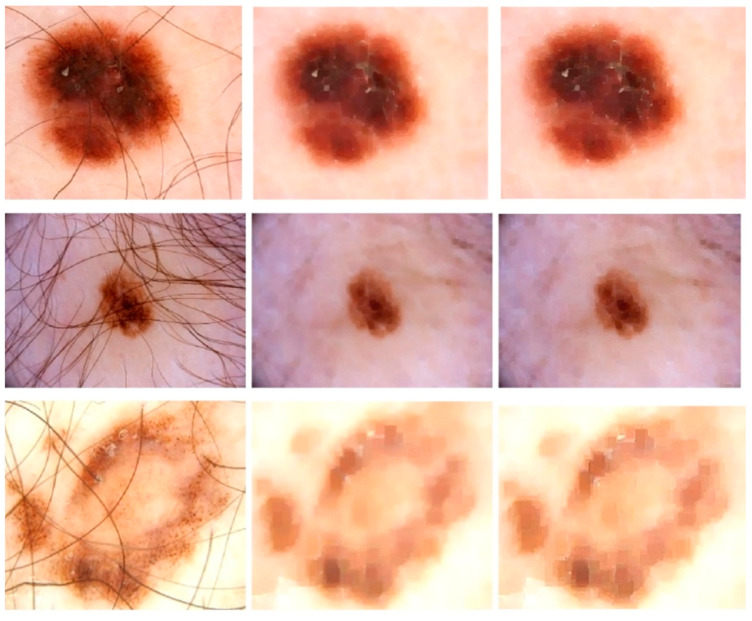
Skin Refinement on ISBI2016 Dataset: Column 1 shows Original Images, Column 2 shows the images after closing morphological operation, and Column 3 shows results after a sharpening kernel is applied.

The initial stage of the proposed model is image enhancement because medical images must be preprocessed before being used as input. This technique also leads to highly accurate segmentation since more sharp and well-contrasted images allow the model to pinpoint the lesions because the lesion may have a very similar visual appearance to normal skin in its early stages. The images are usually in the form of a 2D array or a 3D array of pixels. The color (RGB) image is the 3D array of pixels. Frequently utilized preprocessing approaches include smoothing of the images, resizing, ROI detection, and de-noising. Gaussian Smoothing is a highly recommended approach for removing the artifacts from the images. Along with the Gaussian function, we used Dilation followed by Erosion operation (i.e., Morphological closing) and sharpening kernels in this research study to eliminate hair and air bubbles from images while also increasing image contrast. This procedure dilates a dermoscopic image of skin cancer and afterward erodes that resulting image, employing the same structural element including both processes. Morphological closure is beneficial for refilling small gaps in conjunction with maintaining the size as well as the shape of larger holes in dermoscopic images. Figure 2 shows some sample images that undergo these preprocessing steps, where the first column shows the original input images, the second column is the morphological closing result on images, and the last column shows the result of sharpening.

### 3.2. Data Augmentation

Normally, the training samples that are publicly available for all the classes are not distributed equally, so this will create a class imbalance problem. Second, for deep learning models, an abundance of data will be the ultimate need for improved generalization and performance. The datasets used in this study have a very limited number of samples, specifically DermIS and DermQuest. Hence, in this study, we extend the samples by utilizing different augmentation methods such as flipping, cropping, and rotating at different angles. Table 1 shows these parameters. By applying all these parameters, we generate 15 new samples from a single image. The sole reason we use this step in our framework is to address overfitting issues and improve the model’s performance accuracy.

### 3.3. Melanoma Lesion Segmentation Using cGANS

Generative Adversarial Networks (GANs) are sophisticated deep learning models that come within the domain of generative models. In general, generative models are used to produce data artificially for a variety of objectives. There are several types of GANs such as Wasserstein GANs [65], Conditional GANs [66], Cycle GANs [67], Style-based GANs [68], Progressive GANs [69], etc. However, in this research study, we employ Conditional GANs for the task of melanoma segmentation. The major rationale for using this GAN is to perform lesion segmentation since, in this type of GAN, the data that are generated artificially are conditioned on certain criteria. For instance, if class labels are provided, those can be utilized as input. However, in basic GANs, data samples are generated at random from random noise vectors and there is no method to control the sorts of images produced. Therefore, we formulate the problem of skin lesion segmentation with the help of cGANs. It involves the generation of images with a condition. In the proposed method this condition is a source image, i.e., the dermoscopic image of skin cancer, while the outcome of the model is segmented images in which the lesion is highlighted. This type of image generation is also considered targeted image generation in which both types of CGAN modules, i.e., generator and discriminator, are conditioned on dermoscopic images during training.

#### 3.3.1. Problem Formulation

To meet the contributions presented in this study, we employ the cGANs for melanoma lesion segmentation. As stated previously, we first preprocess the dermoscopic images to eliminate the noise as well as artifacts from the images. Later on, the preprocessed images and their ground truth images are used as inputs to cGANs to generate the segmented images.

The proposed model has two deep learning models, namely, the generator and discriminator. The objective of the generator is to generate the segmented images of skin lesions, while the discriminator classifies whether the generated segmented image of the lesion is real or fake. The weights of the discriminator are updated by themselves without taking the generator into account, while the weight updates of the generator are performed with the help of the discriminator model. Hence, the objective of conditional GANs is given below [66]:

(1) $$L_{C}GAN\;\left(G,\;D\right)=E_{x,y}\;\left[log\left(D\left(x,y\right)\right)\right]+E_{x,z}\;\left[log\;\left(1-D\left(x,G\left(x,z\right)\right)\right)\right]$$

In the above Equation (1), G is the generator and D is the discriminator, z is the random-noise vector, x is the input (i.e., dermoscopic image and real ground truth image of lesion), and y is the generated segmented images of lesions by the generator, and term $L_{C}GAN$ is the loss function of conditional GANs. Moreover, G is for minimization of objective, whereas D is for maximization, i.e., [66],

(2) $$G^{*}=argmin_{G}\;max_{D}\;L_{cGAN}\;\left(G,D\right)$$

In the above Equation (2), $G^{*}$ is the objective of the generator.

#### 3.3.2. Design and Architecture of cGAN

The overall architecture of cGANs consists of two further deep learning models, i.e., discriminator and generator. The proposed discriminator model for classifying the segmented skin lesion images is simply the deep convolutional neural network. The input of this CNN is the dermoscopic image of skin lesion (referred to as the source image) and corresponding segmented ground truth images (referred to as the target image). The output of the model is to estimate the likelihood that the segmented ground truth image is a real or fake translation of the dermoscopic image of the skin cancer. The input dimension of both dermoscopic and ground truth images is set to 256×256×3. More precisely, the discriminator is built over the improved receptive field in which associations among the outcome of the model are related to the total number of pixels in the dermoscopic image. This model is referred to as patch-GAN in which the outcomes are mapped over the patch of 70×70 image provided at input. This module simply penalizes architecture at the patch level. The discriminator attempts to determine if every N×N patch in the segmented ground truth image is real or fake.

Hence, the Patch-GAN-based discriminator first concatenates the two inputs, i.e., dermoscopic image and ground truth image. Later on, the discriminator model consists of five convolution layers having units 64, 128, 256, 512, and 512, respectively. A batch-normalization layer and Leaky ReLu activation function are applied after every convolution layer. The alpha parameter in the Leaky ReLu activation function is set to 0.2. The size of kernels in the discriminator is set to 4 by 4, and the weights of these kernels are initialized with Random Normal with a standard deviation of 0.02. Finally, a 2D convolutional layer is employed that performs the predictions in the form of a patch. This patch output is given to the sigmoid layer, resulting in the label of the image as being real or fake. Following on, the architecture of the generator model is based on UNet including the modules of the encoder and decoder. The input of the generator model is the dermoscopic image (also referred to as the source image) and the output of the model is the segmented image in which the lesion is highlighted as foreground pixels. This can be accomplished by first encoding the image followed by passing the resulting feature maps to the bottleneck layer. Later on, the upsampling path also called the decoder model is employed to upsample the resulting information from the bottleneck path in the form of a segmented image. Skip connections are also inserted among the encoder and decoder modules. The input of the dermoscopic image is passed through seven convolution blocks. Each convolution block consists of one convolution layer followed by batch normalization and leaky ReLu. The kernel size in the convolutional layers is 4 by 4, the stride value is 2, and the weight initialization is Random Normal. There are 7 encoder blocks in total, with a total of 64, 128, 256, 512, 512, 512, and 512 convolutional filters in each block. Similarly, the bottleneck part consists of one convolution layer with the activation function ReLu. This layer’s convolutional filters are similarly 512 with stride value of 2 and kernel size of 4. Following on, there are seven decoder blocks consisting of transposed convolutions as well as batch normalization. A dropout layer of 0.5 is also after batch normalization in the decoder part of the generator model. Finally, a 2D convolutional layer is added to generate 2D segmentation map results or to generate a segmented image in which the skin lesion is highlighted.

#### 3.3.3. Training of cGAN

The discriminator is trained with original ground truth images of skin lesions as well as generated (fake) ground truth images of skin lesions. However, the training of the generator is dependent upon the discriminator. An update of the generator model is undertaken to reduce the loss of discriminator for the segmented skin lesion images that are labeled as real. Through this process, it is expected that the best-quality segmented images will be generated. To reduce the $L_{1}$ loss, we also update the generator, as this loss represents the error between the real and generated segmented images of skin lesions. Therefore, it is logical to state that generator weights are updated with both adversarial and $L_{1}$ loss. The generator model is stacked over the discriminator, resulting in a new model having weights of the other two models. On the other hand, the discriminator weights are adjusted independently. In addition, to test the importance of conditioning the discriminator, an unconditional version wherein the discriminator does not perceive x is also given in Equation (3) [66]:

(3) $$L_{GAN}\left(G,D\right)=E_{y}\;\left[log\left(D\left(y\right)\right)\right]+E_{x,z}\;\left[log\;\left(1-D\left(G\left(x,z\right)\right)\right)\right]$$

In the above Equation (3), G is the generator and D is the discriminator, and term $L_{GAN}$ is the loss function of conditional GANs. Recent methods have shown the significance of integrating the objective of GAN in a traditional loss function, such as distance $L_{2}$ [19]. The job of D is still the same, but the G is tasked not just to fool the D but also to near the ground truth (i.e., segmented lesion images) of the $L_{2}$ sense. We can explore this option by utilizing $L_{1}$ as compared with $L_{2},$ as $L_{1}$ produces low blur [66].

(4) $$L_{L_{1}}\;(G)=E_{x,y,z}\;\left[\|y-G{\left(x,z\right)\|}_{1}\right]$$

The final objective of the model is given in Equation (5) [66]:

(5) $$G^{*}=argmin_{G}\;max_{D}L_{cGAN}\left(G,D\right)+\lambda L_{L_{1}}\left(G\right)$$

**Figure 3:**
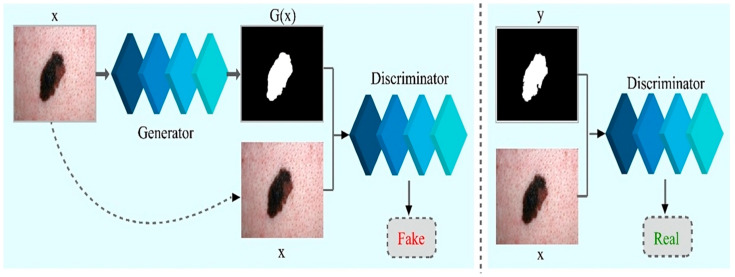
Training of Conditional Generative Adversarial Networks for melanoma lesion segmentation.

In the above Equation (5), the λ is the weight of the loss function and $G^{*}$ is the final objective of the model. The network could still acquire a map from x to y (i.e., dermoscopic image to segmented skin lesion image) without z, but it would not produce deterministic output and, thus, fail to match any distribution other than a delta function. The previous GANs had acknowledged this and provided Gaussian-noise z as an input to the G, with x [53]. In early findings, no efficient approach exists. The G only learns to overlook noise, which is consistent with the study in [52]. As an alternative, noise is given only in form of dropout in the final model, applied on numerous layers of the model for training and testing time as well. Training of CGANs is depicted in Figure 3.

#### 3.3.4. Hyper-Parameters of cGAN Model

The discriminator’s hyperparameters include the loss function, which is binary-cross entropy, a weight optimizer set to Adam, a learning rate of 0.0002, and a β value in the optimizer set to 0.5; in addition, the padding in convolutional layers remains “same” while the weights of the discriminator were initialized with the “Random Normal” method. Similarly, the hyperparameters of the generator model include weight initialization, weight optimizer, and learning rates, which are similar to those of the discriminator. In addition, the proposed algorithm is implemented in Python with a Keras deep learning framework and simulations were carried out on Google Colab with K80 GPU along with 12 GB of RAM. Moreover, the training time for one simulation of CGANs is about 3.5 h approximately.

## 4. Results

In this section, we analyzed and discussed the findings of CGAN-based melanoma image segmentation. We also discussed the evaluation metrics and data used in the study.

### 4.1. Datasets

**Figure 4:**
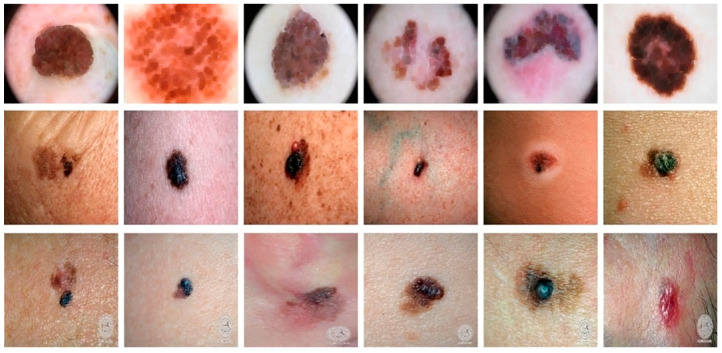
Sample images from the datasets: Horizontally Row 1 shows some sample images from the ISCI2016 dataset. Row 2 shows some sample images from the DermQuest dataset and Row 3 shows some sample images from the DermIS dataset.

To look at the cGAN generality, we tested the approach on three different datasets, i.e., DermIS [70], DermQuest [70], and ISCI2016 [71]. In all these datasets, we had color images. The DermQuest dataset contains a total number of 274 images along with their corresponding ground truth images. Similarly, DermIS datasets contain a total of 69 images and their ground truth images. The total number of image samples in both datasets is quite low; thus, we used data augmentation to increase the number of samples for both of the datasets. Likewise, the dataset ISCI2016 comprised 900 melanoma images for training and 379 testing images. Figure 4 shows the sample images from the datasets.

### 4.2. Evaluation Metrics

To evaluate the performance of the model, we evaluated the datasets utilizing accuracy measures, dice score, specificity, sensitivity, and Jaccard score. The following equations illustrate these measures:

(6) $$\mathrm{Accuracy}\;=\frac{\mathrm{TP}+\mathrm{TN}}{\mathrm{TP}+\mathrm{TN}+\mathrm{FP}+\mathrm{FN}}$$

(7) $$\mathrm{Dice}\;\mathrm{score}\;=\frac{2.\mathrm{TP}}{2.\mathrm{TP}+\;\mathrm{FP}+\mathrm{FN}}$$

(8) $$\mathrm{Specificity}\;=\frac{\mathrm{TP}}{\mathrm{TP}+\mathrm{FP}}$$

(9) $$\mathrm{Sensitivity}\;=\frac{\mathrm{TP}}{\mathrm{TP}+\mathrm{FN}}$$

(10) $$\mathrm{Jaccard}\;\mathrm{score}\;=\frac{\mathrm{TP}}{\mathrm{TP}+\mathrm{FP}+\mathrm{FN}}$$

In the above equations, TP is the value of true positives, FP is the value of false positives, TN is the value of true negatives and FN is the false negative value, and 2 shows the 2 x values of the corresponding value.

### 4.3. Results on DermIS Dataset

**Figure 5:**
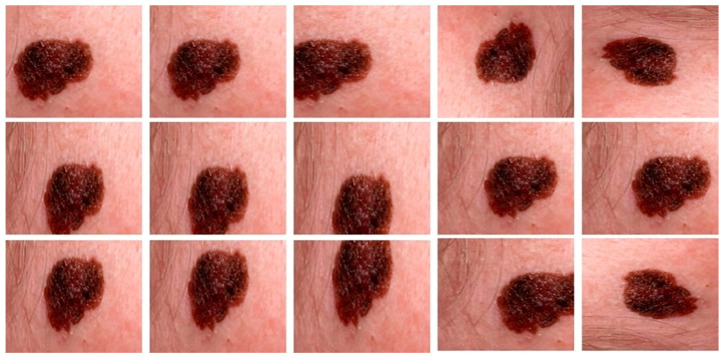
The augmented samples of the images from the DermQuest dataset.

**Figure 6:**
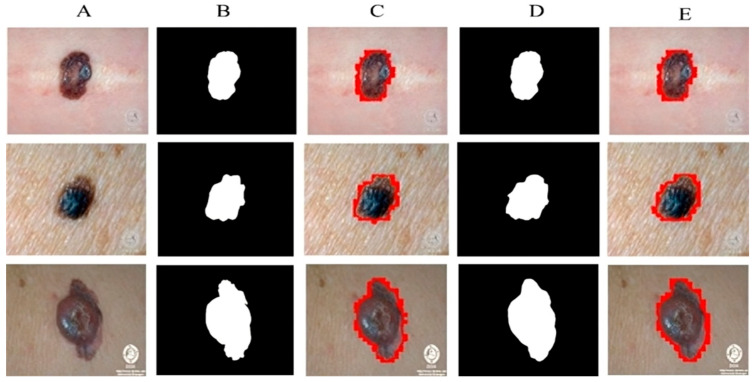
Melanoma segmentation results of DermIS dataset from the skin with the respective masks and contour images.

All experiments are carried out by utilizing melanoma images and their related ground mask images. As previously stated, we used three separate datasets in our research. Artifacts such as hair, air bubbles, and other noise can be seen in the dataset images. The existence of these artifacts has an impact on the outcome of our study. To address this issue, we used several preprocessing steps as well as augmentation methods on just the DermQuest and DermIS datasets. This step is performed to increase the data samples as fewer training samples lead to overfitting and class imbalance issues. Figure 5 shows the image samples after augmentation. For the melanoma lesion segmentation, we utilized the cGAN model. This automatically learns from the input images and provides us with the required melanoma lesion segmentation. Figure 6 shows the segmentation of melanoma on the DermIS dataset from the skin with the respective masks and contour images. In Figure 6, column A shows the preprocessed images of the DermIS dataset. In the next step, column B shows the actual masks of the corresponding input images. Next, column C shows the actual images with a contour. The red dots in column C shows the contour.

The masks that were predicted by the proposed model are shown in column D and the last column E, the sample image that was predicted by the model is shown with a contour mask around their boundaries. Furthermore, as demonstrated in Table 2, the accuracy, dice, Jaccard, and specificity attained on this dataset are 97%, 93%, 97%, and 95%, respectively.

### 4.4. Results on DermQuest Dataset

Similar to the DermIS dataset, all the experiments are conducted by utilizing dermoscopic images and their ground masks. Likewise, in DermIS, we also applied preprocessing steps on this dataset to reduce the artifacts such as hair, air bubbles, and other noise. To improve our model’s performance, we used augmentation steps on this dataset to increase the number of data samples, as fewer training samples lead to overfitting and class imbalance issues. For the lesion segmentation, we utilized the cGAN model. This automatically learns from the input images and provides us with the required melanoma lesion segmentation.

**Figure 7:**
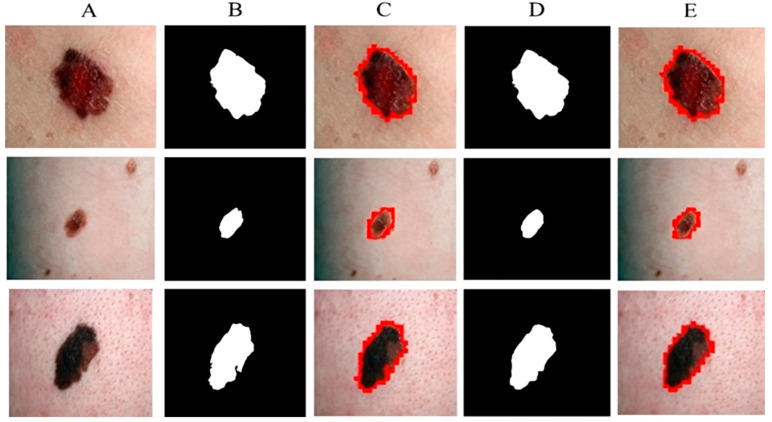
Melanoma segmentation results of DermQuest dataset from the skin with the respective masks and contour images.

**Figure 8:**
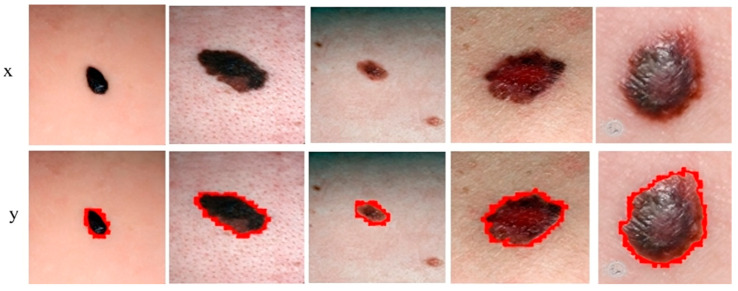
Sample melanoma segmentation results of datasets in form of contour images.

Figure 7 shows the segmentation of melanoma lesions of the DermQuest dataset from the skin with the respective masks and contour images. In Figure 7, column (A) shows the original DermQuest preprocessed images. In the next step, column (B) shows the actual masks of the corresponding input images. Next, column (C) shows the actual images with contour. The red dots in column C shows the contour. The masks that were predicted by our proposed model are shown in column D and the last column E, the sample image that was predicted by our model is shown with a contour mask around its boundaries. Similarly, the accuracy, dice, Jaccard, and specificity achieved on this dataset are 95%, 95%, 95%, and 90%, respectively, as shown in Table 2. Figure 8 shows the sample test image results from all three datasets, i.e., DermIS, DermQuest, and ISCI 2016, in the form of contour images. In Figure 8, the above row x shows the input images while the second row y shows the results in form of contour images.

**Figure 9:**
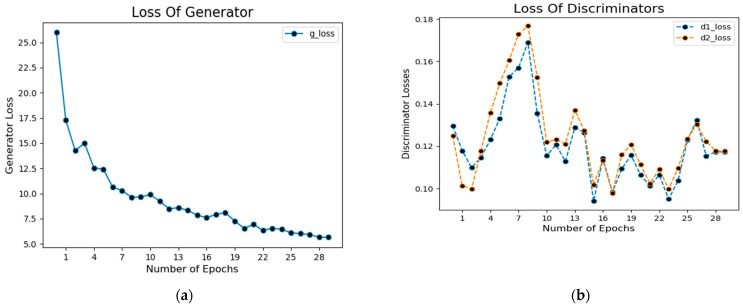
Loss of generator (**a**) and discriminator (**b**) of CGANs model.

In addition, Figure 9a shows the loss of the generator during the training process. The blue dotted lines in this figure represent the generator’s loss.

Similarly, Figure 9b shows the loss value concerning the number of epochs during the training of the network in which dotted blue lines represent discriminator loss on real examples, whereas orange dotted lines show discriminator loss on fake examples (i.e., fake segmented images).

### 4.5. Comparison with ISCI2016 Challenge

The proposed model was also evaluated on a benchmark dataset named ISIC 2016 by “International Symposium on biomedical images (ISBI) in the challenge of Skin lesion analysis towards Melanoma detection”. This dataset contains 1279 images, 900 for training, and 379 for testing. In this ISBI 2016 challenge, nearly 28 groups participated and submitted their results, as shown in Table 3. The ISBI ordered the participants to segment as per their highest average score of Jaccard. The accuracy, dice, Jaccard, and specificity achieved by the proposed model on the ISCI2016 challenge dataset are about 95%, 90%, 95%, and 91%, respectively, as shown in Table 3. From Table 3, it is observed that to reduce the segmentation error, all the participants utilized deep learning because of precise segmentation and effective backpropagation learning. Moreover, the participants of ISBI 2016 also utilized a pre-trained model such as AlexNet [75], VGG16 [76], and ResNet [77] to estimate the boundaries of melanoma. 

In comparison, our technique achieved the best outcomes by outperforming all previous trials. Even though we did not use post-processing to improve segmentation outcomes, the Jaccard score of this study is the greatest among the leading participants. The second top participant in the ISBI challenge is CUMED; they utilized a fully residual network without utilizing any preprocessing and post-processing approaches. We also compared the performances with some traditional methods of segmentation. These types of comparisons on the same ISBI2016 dataset have also been carried out and performed in the existing research studies [15,33]. It is noticeable from Table 2 that these approaches, including Adaptive Thresholding [23], Bootstrap learning [60], Contextual [59], ISO [27], Level set [72], Sparse coding [73], Region growing [31], and FCN [74], give the lower results as compared to the proposed approach. Table 2 presents the comparison between existing techniques with our proposed framework. Besides traditional approaches, we also performed a comparison with deep learning techniques such as FCN. With this method, the Jaccard score is about 86%, which is lower than the proposed method. It was logically concluded that the suggested method is good enough for segmenting the melanoma lesions from given dermoscopy images.

Moreover, if the proposed CGAN model is evaluated in terms of addressing challenges over existing methods then one of the advantages of the proposed CGANS is that it efficiently handles melanoma lesion segmentation in the presence of visual appearance, texture, size, and shape problems; however, traditional methods such as OSTU or thresholding-based segmentation methods fail when some diverse changes are observed in the melanoma lesion images because these are parametric methods that must be set manually. More specifically, the same threshold criterion may not be applicable for all sorts of dermoscopic images having contrast and sharpness issues. The proposed CGAN model does not require any post-processing steps or any threshold for every specific dermoscopic image. CGANs are generative deep learning models utilizing convolution and max-pool layers to enable automated feature learning. These layer-level configurations in the discriminator and generator models are first utilized to extract the features, which are then utilized to generate the required segmentation maps. These layers hold the properties of scale invariance, preserve neighborhood semantics with the help of shared parameters among kernels, and capture different receptive fields using different size kernels. The feature learning process starts from low-level features to high-level features including learning edges, edges to shapes, and shapes to structures. This kind of automated feature learning will handle variability in object size, e.g., in our case, lesion size, shape, and color. Second, because CGANs are a generative class of deep learning models, their distinct learning or training technique will provide an additional benefit in accurate melanoma segmentation. The objective of the discriminator model in CGANs is to classify the real and fake segmented images (i.e., generated by the generator model); however, the generator model generates images that are closer to real images based on the feedback of the discriminator. The generator model is updated to decrease discriminator loss for segmented skin lesion images that are labeled as real. The final objective given in Equation (5) will be able to train the discriminator and generator model in a zero-sum game, and based on this, the generator model will generate more accurate segmentation maps for skin lesions in the presence of variability in lesion size, shape, and color-related problems. Therefore, the strength of the proposed CGAN model is that it can handle these challenges and segment the skin lesions in more precise localizations. The suggested model is based on deep learning, which is a more generalizable and cutting-edge way than conventional classic methods such as active contour, level set, Otsu, and so on. The comparison study in Table 2 and Table 3 demonstrates that the suggested model is more robust and performs well on all performance metrics, including accuracy, dice score, Jaccard, sensitivity, and specificity. The suggested model’s scalability includes the ability to be extended to other forms of medical imaging tasks, such as semantic segmentation of different kinds of cancers, such as blood cancer, in which blood cells are segmented for medical image analysis. Similarly, it may be extended to perform data augmentation in order to address concerns of class imbalance and dataset scarcity. On the other hand, it is essential to discuss the limitations of the model for further future research. As a result, one limitation of the model is that given a skin lesion, it can precisely localize the skin lesion; hence, it may be beneficial if, in addition to localization, the model outputs the category of skin lesion, such as melanoma’s classes or the severity of the lesions. However, because the suggested CGANs are based on generative models, an additional model that classifies the localized segmented images is required to undertake a more comprehensive analysis of dermoscopic images. Second, assessing CGAN performance as cross-data set validation is critical for determining generalizability.

Furthermore, if the suggested model is examined to see how it might be used in real-world clinical settings, various important aspects emerge. For example, in order to deploy it in a real-world scenario, the model must first be converted to deployable APIs. Second, a system with strong computational capabilities, such as GPUs, RAM, and other resources, is required for smooth operation. However, establishing IoMT-assisted medical healthcare systems presents a variety of challenges. These challenges include security threats, cost, and other administrative challenges. The sensors might be faulty sometimes; thus, an appropriate methodology for validating the data collected from the sensors is required. To guarantee that data are gathered effectively, as well as sensors offering reliable information, the IoMT system must adopt a high degree of precision. Moreover, the misinterpretation of data as well as information resulting from devices also leads to adding a limit to the IoMT-based healthcare systems. From the perspective of the proposed model, as well as other CAD solutions for different diseases, it is logical to say that such a system cannot completely replace doctors but can work as an assistant for the doctor, as well as provide advantages to patients in remote locations.

## 5. Conclusions

In this paper, we suggested an approach for segmenting melanoma lesions using Conditional Generative Adversarial Networks. The proposed method is appropriate for accurately segmenting the lesion. As in melanoma lesion segmentation, the normal and affected regions are visually the same, so this approach learns the loss function, as well as maps input images to corresponding output images, for accurate segmentation. The proposed technique efficiently detects melanoma lesions as compared to the state-of-the-art approaches. This cGAN network automatically learns the loss function and this characteristic makes it applicable to many other medical image segmentation problems. We have attained higher performance accuracy for the DermIS and DermQuest datasets, i.e., 99% and 97%, respectively. While the challenging dataset ISCI 2016 also produces the greatest results, i.e., 95% performance accuracy. Future work will entail improving the cGAN model and adding more challenging images, as well as increasing the number of samples in the training data. In the future, we hope to analyze particular color images rather than RGB color spaces.

## Figures and Tables

**Table 1:** Types of Data Augmentation.

Sr. No	Augmentation Steps	Parameters
1	Rotate	90°, 180°, 270°
2	Crop from Right	45°, 60°, 90°
3	Crop from Left	45°, 60°, 90°
4	Crop from Top	45°, 60°, 90°
5	Crop from Bottom	45°, 60°, 90°
6	Flipping	Left Right
7	Shifting	shifted by (25, 25) pixels

**Table 2:** Comparison of state-of-the-art approaches with our proposed model.

Techniques	Accuracy	Dice Score	Jaccard	Specificity
Adaptive Thresholding [23]	72%	56%	45%	80%
Bootstrap learning [60]	78%	72%	57%	75%
Contextual [59]	83%	75%	60%	77%
ISO [27]	82%	68%	56%	78%
Level set [72]	70%	58%	46%	79%
Sparse coding [73]	91%	80%	66%	86%
Region growing [31]	73%	55%	43%	76%
FCN [74]	82%	82%	86%	70%
Proposed (DermQuest)	99%	95%	99%	90%
Proposed (DermIS)	97%	93%	97%	95%
Proposed (ISCI2016)	95%	90%	95%	91%

**Table 3:** Comparison of proposed model with ISBI2016 challenge winners.

Technique	Accuracy	Dice Score	Jaccard	Sensitivity	Specificity
ExB	95%	91%	84%	91%	96.5%
CUMED	94%	89.7%	82.9%	91.1%	95.7%
Mahmudur	95.2%	89.5%	82.2%	88%	96.9%
SFU-mial	94.4%	88.5%	81.1%	91.5%	95.5%
TMUteam	94.6%	88.8%	81%	83.2%	98.7%
Uit-Seg	93.9%	88.1%	80.6%	86.3%	97.4%
IHPC-CS	93.8%	87.9%	79.9%	91%	94.1%
UNIST	94%	86.7%	79.7%	87.6%	95.4%
JoseLuis	93.4%	86.9%	79.1%	87%	97.8%
Marcoromelli	93.6%	86.4%	78.6%	88.3%	96.2%
Proposed	95%	90%	95%	91%	90%

## Data Availability

The data used in this work are publicly available and can also be accessed by contacting the corresponding author.
